# A Memoir on the Nutrition of the Foetus

**Published:** 1803-03-01

**Authors:** I. F. Lobstiere

**Affiliations:** the Medical School of Strasburgh, Member of Several Societies, &c.


*A* MEMOIR *on the* NUTRITION *of the* FOETUS, *by* I. F. LOBSTIERE, *M. D. of the Medical School of Strasburgh, Member of several Societies, &c.; communicated by our Correspondent at Paris.*

WITH a view to elucidate many of the abstruse points which relate to the nutrition of the fœtus, Cit. Lobstiere has occupyed himself, particularly for some years, on this subject. The important situation which he fills in the school of Strasburgh, has given him frequent opportunities of observing the various changes which the viscera undergo dergo during the period of gestation; he has been frequently able to verify what anatomists have already remarked of the disposition of the membranes of the placenta, and he has carried his enquiries into the state of other animals during this period, with a view to ascertain the relation which exists between the placenta and matrix, in order to draw a comparison between them and the human subject. In the examination of this question, two objects principally presented themselves to the author. The first is, to determine the nature, origin, and essential qualities of the fluid necessary to the nutrition of the fœtus. The second, to shew how this matter is conveyed to its destination. These two questions, though intimately connected in their nature, still establish the ground of decision of the Memoir which the author now offers to the public. The first part contains anatomical and physiological remarks on the membranes of the ovum and placenta, considered as the organs which furnish the matter of nourishment. The second is employed in the consideration of nutrition properly so called, or the passage of the matter of nourishment into the body of the fœtus. To determine how this matter is converted into the proper substance of the individual, does not enter into the author's plan. In the first part of this work, the author treats of the ovum and its membranes; he first gives a description of the decidua, and indicates its relations with the uterus and ovum; he examines its appearances through the microscope, and gives a history of its organization in the cow and sheep; he next enquires, whether or not it exists in the matrix in the unimpregnated state, whence its origin, and in what it differs from the false membranes? He investigates the structure of the chorion, he discusses the question of its vascularity, and how far it is analogous to other diaphanous exhaling membranes, and points out its connexion with the placenta. In speaking of the amnios and its structure, the author seems to think, that the existence of a space filled with water, as many anatomists have thought, is not fully demonstrated, and that when these waters are found they are the effect of disease, and not a natural appearance. The vital powers of the membranes of the ovum, as well as the question whether or not they are derived from the mother or fœtus, engage his enquiry; he shews the differences which exist in this respect in different kinds of animals, and looks on it as highly probable the opinion which gives to these membranes the same powers as diaphonous membranes in other parts of the body, and shews their functions to to be the same as other exhaling membranes. He enquires into the source of the liquor amnii, and he explains by those facts already ascertained, why this water is accumulated, and how it is renewed. Speaking on this point, he makes a digression on the origin of the caseous matter covering the skin of the fœtus, and thinks, contrary to the generally received opinion, that this is a secretion made by the skin of the fœtus at a certain period of gestation. He draws, at length, some physiological consequences from the known disposition of the membranes of the ovum. The existence of the alantois has been admitted by some anatomists, and contested by others. The author thinks, however, that, "if we reflect on the light thrown on the structure of the ovum by modern enquirers during the first periods of pregnancy, it is probable that those anatomists who have described an alantois in the human subject, are not wrong; nay, that it is probable that it is to this part they have given the name of vesicula umbilicalis." As this latter is as yet but little known, and has not met with all the attention it deserves, the author details a number of observations that have been made on this body, and adds some curious remarks, the fruit of his own experience. According to him, there exists the most complete analogy between the vesicula umbilicalis and alantois, in birds and quadrupeds; and to support this analogy, he enters into some details in comparative anatomy, from which he concludes, that the use of the alantois is not, as has been supposed hitherto, a reservoir of urine. The author next describes the placenta at the different periods of pregnancy. In describing the spongy membrane which covers the uterine surface of the placenta, he shews that the existence of this membrane does not date from the earliest periods of gestation. The structure, connexions, and uses of the parts engage a considerable portion of the author's attention, and he refutes the opinion of the anastomosis of the vessels of the uterus and placenta; considering the opinions relative to the passage of the blood from the mother to the infant by absorption, he asserts that this absorption does not take place at every period of pregnancy, and that the valvular vessels, such as Reuss has described them, do not exist in the placenta. According to our author, the connexion of the uterus and placenta are not the same throughout the state of pregnancy, but that it differs at different periods. This first part is terminated by researches on the umbilical cord; and he quotes the experiments of Hunter on the contractility of these vessels, and concludes by observing that that the vital powers of the placenta are derived from the mother.

In the second part, the author treats of the nutrition of the fœtus, the opinions on which he discusses in the three following questions. 1st. Is the fœtus nourished by the liquor amnii? 2d. Does it draw its nourishment from the placenta? Or, 3dly, Are these two sources united at one and the same time, in affording the matter of increase? And if in the first case, is the matter taken in by the mouth or absorbed by lymphatics of the surface? After giving the various opinions in support of these several questions, the author gives his own reasons for thinking the fœtus nourished by the liquor amnii, and taken in by cutaneous absorption. He here digresses, by enquiring into the nature of the caseous matter covering the surface of the fœtus; he gives his ideas on the sebaceous glands of the skin, which he considers as the source of this secretion. He undertakes to shew that the structure and uses of the placenta differ according to the period of gestation, and he has recourse both to human and comparative anatomy, to shew those changes which take place in the earlier periods. The author establishes an analogy between the structure of the cotyledons of quadrupeds, the umbilical vessels of the chick, and the human placenta; and he shews from observations which he has made, that the umbilical veins are formed before the umbilical arteries, that in so much as the veins are the only vessels of the placenta, they perform the function of absorption, and that they convey a milky fluid which is thrown out during the first periods of pregnancy, between the matrix and placenta.

A French reviewer, who reviews these opinions as highly probable, observes, that by adopting these observations of Cit. Lobstiere, we may explain, 1st, The facility with which the umbilical vessels degenerate into hydatid vesiculæ. 2d. Why the vessels of the placenta, even those of the fœtus, have been injected from those of the matrix. The author shews afterwards, from analogy, that the umbilical arteries, though formed after the veins, yet anastamose with these vessels. After giving the various opinions on the functions of the placenta during the latter periods of pregnancy, the author draws a parallel between the functions of the lungs and those of the placenta; he asserts that the blood acquires a new stimulating quality in the placenta, and points out the analogy which he conceives to exist between the pulmonary and placental circulation. C. Lobstiere thinks that the stimulus acquired in the placenta is caloric; and he examines the question why the temperature of the fœtus is less than that of the mother? Why the heart in the fœtus is stimulated in the first periods of pregnancy? and whether or not the fœtal blood undergoes any depuration in the placenta? It appears to our author probable that this depuration takes place in the liver, in the intestines, and even in the cutaneous organs. With respect to the vesicula umbilicalis, he thinks that it is subservient to the nutrition of the embryo, and he compares it to the cotyledons of plants; he even thinks that the albuminous matter of the umbilical cord is a source of nourishment to the fœtus. After pointing out the reasons why the growth of the fœtus in all animals is slower towards the latter period, he concludes his work by some considerations on the causes which lead to partution, and on those which produce the separation of the placenta. With respect to the first, he thinks that we should not attribute the expulsion of the human fœtus wholly to that particular disposition of the matrix, by which its fibres, as well those of the body as of the neck of the womb, and which through the previous periods are in a state of antagonization, become so affected as that, at the approach of labour, the fibres of the body become more powerful than those of the neck; but he thinks, though this opinion may be true, that yet we should admit as an accessary cause, a power existing in the fœtus itself, which no longer finding a sufficient quantity of matter of nourishment, exerts itself to quit the matrix, and by this means excites it to contraction. "The fœtus in this case, says the author, does no more than a plant, which turns from an ungrateful soil, and looks for one more favourable. It is, he says, no more than we see in birds. The chick breaks the shell which confines it, because its nutrition is exhausted, and because its confined situation is no longer commodious." This work is accompanied by two engravings, which render it more intelligible and interesting. The first plate represents an ovum at the 50th day of pregnancy; the membranes are divided, in order to shew the embryo and vesicula umbilicalis. The second plate shews the vascularity of a portion of a full grown placenta; the vessels are injected.

In giving this account of Cit. Lobstiere's work, we have rigidly abstained from every remark or criticism on the several facts and opinions which he advances; many of which have been already the subject of much disputation, and which we think yet open for discussion. To investigate the opinions of any author who writes writes on a subject of this nature, he should be read. It is impossible to give in any extract of this kind, all the grounds on which he advances his opinions; and it is unfair to seize on the weak point of a work, or the hazarded opinion of an author, to discredit a great deal of useful matter. For these reasons we have confined ourselves to an analysis of the work of Cit. Lobstiere, or rather we have offered the table of contents of his book, in order that those whom the subject interests should know how far the author merits their further consideration. This mode of reporting continental productions will be strictly observed in all future communications of this nature.

